# Supplementary material for: MetaRibo-Seq measures translation in microbiomes
Source: Nat Commun. 2020 Jun 29;11:3268. doi: 10.1038/s41467-020-17081-z (PMC7324362; doi:10.1038/s41467-020-17081-z)
Supplement: Supplementary file 10 — Supplementary Data 7 [file 41467_2020_17081_MOESM10_ESM.zip › File2/Confidence_High_Taxonomy/224088_out.krona.html]

Javascript must be enabled to view this page.

members
magnitude
magnitudeUnassigned
count
unassigned
taxon
rank

224088\_out

2

superkingdom
2
2

phylum
1239
2

2
186801
class

2
186802
order

1671649
2
species

SRS015190\_contig\_number\_contig-100\_14805.56433SRS019685\_contig\_number\_contig-100\_11482.31460
